# Determinants of Health-Related Quality of Life of Epilepsy Patients in Jeddah, Saudi Arabia

**DOI:** 10.7759/cureus.24118

**Published:** 2022-04-13

**Authors:** Haythum O Tayeb, Yousef Alsawwaf, Abeer A Khoja, Bashaer Batarfi, Abrar Baduwailan, Mawaddah Yousef, Reem Aldaheri, Suhailah E Alaklouk, Sarah A AlOthman, Yara Alqurashi

**Affiliations:** 1 Neurology, King Abdulaziz University Faculty of Medicine, Jeddah, SAU; 2 Faculty of Medicine in Rabigh, King Abdulaziz University, Jeddah, SAU; 3 Faculty of Medicine, King Abdulaziz University, Jeddah, SAU

**Keywords:** epilepsy, health-related quality of life (hrqol), patients with epilepsy (pwe), quality of life in epilepsy-31 (qolie-31), saudi arabia

## Abstract

Background

Epilepsy puts an enormous burden on the physical and mental health of patients and can negatively impact their Health-Related Quality of Life (HRQoL). Previous studies have identified multiple factors impacting patients’ HRQoL; however, a consensus has not been reached, as these factors vary among different populations. This has not been sufficiently investigated in Saudi Arabia. Thus, this study aims to assess the HRQoL of epilepsy patients, as measured by the Quality Of Life In Epilepsy-31 (QOLIE-31) questionnaire, to determine the effects of demographics, disease characteristics, and antiepileptic drugs (AEDs) on patients in Jeddah, Saudi Arabia.

Methods

This was a cross-sectional, questionnaire-based study of adult epilepsy patients receiving AEDs who followed up at the epilepsy clinic at King Abdulaziz University Hospital between April 2018 and June 2018. Recruited individuals participated by phone interview.

Results

A total of 200 participants fulfilled our inclusion criteria and consented to participate; 50.0% were male. The average age was 32.8 years. The total average score on the QOLIE-31 was 61.56 (±17.52). QOLIE-31 scores correlated inversely with seizure frequency (p<.000) while the class of medication used, and the number of drugs administered did not correlate with HRQoL.

Conclusion

Whereas previous work has suggested a better quality of life when using newer generation AEDs, our study found no significant difference between the class of medication and whether monotherapy or polytherapy is used. Our findings suggest that efforts to improve HRQoL should be directed toward proper control of seizures regardless of medication class, as the frequency of attacks has the most detrimental effect on patients’ quality of life.

## Introduction

Epilepsy is a chronic disease of the central nervous system affecting millions of individuals worldwide. It alters electrical and chemical processes in the brain, leading to seizures, unusual behavior, and, in multiple instances, loss of consciousness [1]. The worldwide prevalence of epilepsy is estimated to be more than 50 million cases in all age groups [2]. ​In Saudi Arabia, there are 6.5 epilepsy patients per 1000 people, amounting to a total of more than 200,000 individuals [3].

Patients with epilepsy (PWE) face various health, social, and drug-related risks. When left without treatment, patients face increased susceptibility to developing status epilepticus or even sudden unexpected death in epilepsy. Socially, the burden of epilepsy affects patients’ education, employability, and overall public engagement. This is mainly due to the unpredictability of seizure attacks and the social stigma associated with seizures. Additionally, patients are exposed to potentially hazardous antiepileptic drugs [4]. To properly manage epilepsy and control seizures, it is paramount carefully monitor antiepileptic drug (AED) use and adverse effects in addition to periodically evaluating patients' Health-Related Quality of Life (HRQoL) as an outcome measure [5]. While a single AED is effective in controlling attacks in at least half of patients, many individuals fail all medical treatments or require polytherapy to augment seizure control [6]. Moreover, frequent attacks contribute to worsening quality of life (QoL) among patients. This was reported by Norsa’adah et al. who concluded that seizure frequency had the most significant impact on HRQoL [7], which was also reported in the systematic review by Taylor et al. [8].

Some studies have suggested that PWE receiving newer-generation AEDs have higher HRQoL while others indicate that PWE on monotherapy regimens have higher QoL than those on polytherapy. However, there is no consensus on these findings, and a review by Taylor et al. found no clear association between HRQoL and AED regimen [8].

Because epilepsy has varying factors, which can negatively impact HRQoL, and due to the lack of consensus on them, this study aims to determine the effects of disease characteristics (including seizure type, attack frequency, number of AEDs used, and medication class) on the quality of life of PWE treated at a specialized epilepsy unit within King Abdulaziz University Hospital, Jeddah, Kingdom of Saudi Arabia.

## Materials and methods

This was a cross-sectional, questionnaire-based study of adult patients between the ages of 18 and 65 receiving AEDs who followed up at the epilepsy clinic at King Abdulaziz University Hospital between April 2018 and June 2018. Using a convenient sampling approach, 530 patients were contacted by phone to recruit them for the study, from which 200 patients agreed to participate. Exclusion criteria were the following: (a) patients with psychosis; (b) patients with communication barriers (e.g., aphasia); (c) patients not being treated with an AED; (d) patients with intellectual disability; (e) patients with incorrect contact information on our database; (f) patients who refused to participate; and (g) patients who did not respond.

Data collection was conducted by phone interview using a questionnaire surveying sociodemographic information, seizure type, seizure frequency, medications, and social history. Subsequently, the Quality Of Life In Epilepsy-31 (QOLIE-31) questionnaire was administered over the phone. The QOLIE-31 is a standardized, widely used tool that measures QoL in PWE [9]. The QOLIE-31 consists of seven subscales that measure HRQoL domains: seizure worry, overall functioning, emotional wellbeing, energy/fatigue, cognitive functioning, medication effects, and social functioning. The questionnaire is available in multiple languages, including Arabic [10].

Data analysis was performed using the Statistical Package of Social Sciences (SPSS) version 21.0 (IBM Corp., Armonk, NY). Frequencies, percentages, and chi-square were used to analyze nominal variables. Independent Pearson’s correlation coefficient, t-test, and one-way analysis of variance (ANOVA) were used to compare means of QoL between groups of interest. Linear regression analysis was performed using QOLIE-31 total scores as dependent variables.

This study was approved by the Ethics Review Committee at King Abdulaziz University Faculty of Medicine under reference number 525-21.

## Results

Table 1 describes the sociodemographic and basic characteristics of the study sample. The average age of the sample was 32.59 years (SD=10.66) with the ages ranging between 18 and 62 years. The proportion of PWE who had generalized convulsions was 75.7%. The mean total score of the sample on the QOLIE-31 was 61.56 (SD=17.5), whereas the mean t-score was 48 (SD=11.3). The internal reliability of the QOLIE-31 was high (Cronbach’s alpha=0.77). The scores of the QOLIE-31 subscales are reported in Table 2.

**Table 1:** Basic sociodemographic and epilepsy-related characteristics of the study sample as well as mean QOLIE-31 total scores associated with each variable n: Number, QOLIE-31: Quality Of Life In Epilepsy-31 (QOLIE-31)

Variable	n (%)	Mean QOLIE-31 total score
Gender		
Male	100 (50%)	62.61 ± 16.860
Female	100 (50%)	60.51 ± 18.183
Marital status		
Single	116 (58%)	59.43 ± 18.197
Married	84 (42%)	64.51 ±16.190
Living arrangement		
Alone	13 (6.5%)	63.37 ± 16.900
With others	187 (93.5%)	61.43 ± 17.601
Education		
Less than high school	44 (22%)	53.65 ± 21.706
High school	71 (35.5%)	60.85 ± 16.274
College	81 (40.5%)	66.03 ± 13.615
Postgraduate or higher	4 (2%)	70.59 ± 30.188
Job		
Student	99 (49.5%)	58.04 ± 19.318
Employed	37 (18.5%)	63.16 ± 15.727
Unemployed	64 (32%)	66.08 ± 14.359
Income		
<5000 SAR per month	129 (64.5%)	60.41 ± 19.013
5000-10000 per month	41 (20.5%)	62.70 ± 12.828
>10000 SAR per month	30 (15%)	64.96 ± 16.264
Other chronic illnesses		
Yes	154 (77%)	62.10 ± 17.086
No	46 (23%)	59.75 ± 18.993
Seizure types		
Convulsive	151 (75.5%)	60.95 ± 17.731
Non-convulsive	49 (24.5%)	63.43 ± 16.898
Seizure Frequency		
Less than 1 seizure / month	128 (64%)	64.58 ± 16.440
1-4 seizures/ month	41 (20.5%)	61.76 ± 16.399
Weekly seizures	31 (15.5%)	48.82 ± 18.087
Medications		
Monotherapy	116 (58%)	62.46 ± 17.042
Polytherapy (using 2 medications)	67 (33.5%)	61.69 ± 17.034
Polytherapy (using 3 medications)	13 (6.5%)	54.66 ± 21.249
Polytherapy (using 4 medications)	4 (2%)	55.72 ± 27.543
Levetiracetam	139 (69.5%)	61.66 ± 17.752
Carbamazepine	23 (11.5%)	63.18 ± 18.309
Topiramate	6 (3%)	61.73 ± 23.044
Sodium valproate	18 (9%)	56.72 ± 14.793
Phenytoin	5 (2.5%)	58.84 ± 18.003
Lamotrigine	7 (3.5%)	64.81 ± 16.111
Others	2 (1%)	74.5 (SD not reported; n=2)

**Table 2:** The scores of the QOLIE-31 subscales QOLIE-31: Quality of Life in Epilepsy Scale-31, SD: standard deviation

Subscales of QOLIE-31	Mean	SD		Correlation coefficient		P-value
Seizure worry	47.26	24.83		0.632		< .01
Overall functioning	69.66	20.94		0.672		< .01
Emotional well-being	63.60	20.08		0.756		< .01
Energy/fatigue	55.30	22.99		0.651		< .01
Cognitive functioning	59.99	26.96		0.854		< .01
Medication effects	60.99	32.16		0.523		< .01
Social functioning	65.82	22.34		0.747		< .01
Total score	61.56	17.52				

The independent t-test and ANOVA were run to check for differences between demographic and seizure-related subgroups. Being female was associated with a lower mean energy score (p=.009) but not significantly lower total scores. Conversely, total QOLIE-31 scores were higher for participants who were married (p=.043), employed (p=0.013), or had attained higher educational levels (p=.001).

When investigating disease characteristics in relation to QOLIE-31 score, independent t-test, and ANOVA revealed a significant inverse correlation of seizure frequency with lower QOLIE-31 total scores (p<0.000) in addition to lower scores in all seven subscales (p<0.04) except medication effects. There were no significant differences in QOLIE-31 scores between PWE on different AEDs (including levetiracetam, lamotrigine, phenytoin, valproic acid, topiramate, and carbamazepine) nor were there any significant differences between these groups in their mean scores on the subgroups of the QOLIE-31.

We then ran a regression analysis to predict the QOLIE-31 total score as the dependent variable using age, gender, marital status, job status, educational level, number of medications, and seizure frequency as independent variables. The model was statistically significant (R-squared=0.164, F=6.3, p<.007). Age, gender, employment status, and marital status dropped out of significance in the model, whereas educational level and seizure frequency remained significant predictors of QOLIE-31 scores.

## Discussion

This study utilized the QOLIE-31 questionnaire, the most widely used tool to examine the QoL in PWE [8]. The participants were followed at a specialized epilepsy clinic in Jeddah, Saudi Arabia. Similar work was done in Qassim (in the central region of Saudi Arabia) [11], yet this is the first such study from a specialized epilepsy clinic, the first in the Western region of Saudi Arabia, and the second study using the QOLIE-31 in the country.

Looking at the QOLIE-31 survey results generally, the measured mean total score for our patients was 61.56 (±17.5), which is similar to the average result of 58.0 (±13.0) reported in Uganda [12]. We then compared the HRQoL total scores in other countries and found that our mean overall score was higher than the scores reported in Australia 52.9 (±23.1), Benin 52.1 (±33.4), Russia 42.13 (±4.14), and Togo 49.5 (±14.4) [13-15]. On the other hand, the average total in our study was lower than those reported in India and Malaysia with a mean of 74.9 (±20.6) and 68.9 (±15.9) respectively [5,7].

The questionnaire results are comparable to those reported from Qassim where the total score was 64.23 (±17.8) [11]. However, unlike their conclusion that employment status, seizure type, and the number of administered AEDs are the most important factors affecting QoL, in this study, seizure frequency (p<.000) and educational levels (p=.001) were the only independent factors found to be significantly associated with HRQoL.

Males in our sample appeared to have higher QOLIE-31 scores, but this effect was no longer significant when we accounted for confounders. The only significant difference between genders within our data was that female PWE had lower energy scores when compared to male PWE (p=.009).

When clinical characteristics of epilepsy were assessed in relation to HRQoL, we found no statistically significant correlation between HRQoL and seizure type or underlying etiology. In contrast, seizure frequency was detrimental to overall HRQoL (p<0.000). Essentially, the fewer attacks the patient has, the better their QoL will be. This effect is in line with the findings of the systematic review by Taylor et al. [8].

Within the studied population, we found no direct correlation between the number or types of AEDs administered and HRQoL. This is in contrast to findings from previous work that concluded that PWE receiving newer AEDs have better HRQoL [8]. Moreover, we found that the utilization of AEDs, whether monotherapy or polytherapy, showed no significant effect on HRQoL. These are key findings in our study, as they minimize the concerns about QoL when choosing a medication, whether of the older or newer generation, or when using more than one AED.

Limitations and recommendations

Our study was cross-sectional, covered a relatively short period (three months), and depended on recorded PWE contact information stored in our tertiary hospital records, which turned out to be out of date in many cases. This resulted in a fairly limited sample size to generalize the findings. Therefore, we recommend that future studies be conducted prospectively over longer periods with the study team following the PWE directly in the clinic.

## Conclusions

While previous work has suggested a better quality of life when using newer generation AEDs, our study found no significant difference between the class of medication and whether monotherapy or polytherapy is used. Our findings suggest that efforts at improving Health-Related Quality of Life (HRQoL) should be directed toward proper control of seizures regardless of medication class, as the frequency of attacks has the most detrimental effect on the patients' quality of life.
