# Correction: Determinants of Health-Related Quality of Life of Epilepsy Patients in Jeddah, Saudi Arabia

**DOI:** 10.7759/cureus.c423

**Published:** 2026-04-09

**Authors:** Haythum O Tayeb, Yousef Alsawwaf, Abeer A Khoja, Bashaer Batarfi, Abrar Baduwailan, Mawaddah Yousef, Reem Aldaheri, Suhailah E Alaklouk, Sarah A AlOthman, Yara Alqurashi

**Affiliations:** 1 Neurology, King Abdulaziz University Faculty of Medicine, Jeddah, SAU; 2 Faculty of Medicine in Rabigh, King Abdulaziz University, Jeddah, SAU; 3 Faculty of Medicine, King Abdulaziz University, Jeddah, SAU

This article has been corrected to correct several typos and add more detail to the IRB approval statement. In-text corrections:

Abstract – Results

Original: “A total of 200 participants fulfilled our inclusion criteria and consented to participate; 57.4% were males.”

Corrected: "A total of 200 participants fulfilled our inclusion criteria and consented to participate; 50.0% were male."

Materials and Methods

Original: “This was a cross-sectional, questionnaire-based study of adult patients between the ages of 16 and 65 receiving AEDs who followed up at the epilepsy clinic at King Abdulaziz University Hospital between April 2018 and June 2018.”

Corrected: "This was a cross-sectional, questionnaire-based study of adult patients between the ages of 18 and 65 receiving AEDs who followed up at the epilepsy clinic at King Abdulaziz University Hospital between April 2018 and June 2018."

Results

Original: “Table 1 describes the sociodemographic and basic characteristics of the study sample. The average age of the sample was 32.59 years (SD=10.66) with the ages ranging between 16 and 62 years.”

Corrected: "Table 1 describes the sociodemographic and basic characteristics of the study sample. The average age of the sample was 32.59 years (SD=10.66) with the ages ranging between 18 and 62 years."

Table 1 – Other chronic illnesses

Original: “No 64 (23%) 59.75 ± 18.993”

Corrected: "No 46 (23%) 59.75 ± 18.993"

Table 1 – Others medication row

Original: “Others 2 (1%) 74.5”

Corrected: "Others 2 (1%) 74.5 (SD not reported; n=2)"

Limitations and recommendations

Original: “Our study was conducted retrospectively, covering a relatively short period (three months), and depended on recorded PWE’s contact information stored in our tertiary hospital records, which turned out to be out of date in many cases.”

Corrected: "Our study was cross-sectional, covered a relatively short period (three months), and depended on recorded PWE contact information stored in our tertiary hospital records, which turned out to be out of date in many cases."

